# Assessing Online Flow Across Cultures: A Two-Fold Measurement Invariance Study

**DOI:** 10.3389/fpsyg.2019.00407

**Published:** 2019-03-15

**Authors:** Elwin Hu, Vasileios Stavropoulos, Alastair Anderson, Michael Clarke, Charlotte Beard, Stelios Papapetrou, Rapson Gomez

**Affiliations:** ^1^School of Psychology, Counselling and Psychotherapy, Cairnmillar Institute, Hawthorn East, VIC, Australia; ^2^Department of Psychology, Palo Alto University, Palo Alto, CA, United States; ^3^Cyprus Youth Organization, Nicosia, Cyprus; ^4^School of Health and Life Sciences, Federation University, Mount Helen, VIC, Australia

**Keywords:** Flow, online Flow, Online Flow Questionnaire, measurement invariance analysis, psychometrics, psychometric consistency, culture

## Abstract

The association between online Flow and Internet Gaming Disorder (IGD) has attracted significant attention. Despite the consensus that online Flow plays a pivotal role in the development of IGD and other Internet addictive behaviors, there has been a lack of consistency in measurement scales used to assess online Flow. Even widely used measures of online Flow have not been psychometrically assessed across culturally diverse populations of gamers. Such an assessment would enhance the accuracy of cross-cultural comparisons. Attending to this need, the present study assessed the psychometric properties of the binary coded (i.e., Yes, No) Online Flow Questionnaire (OFQ), while concurrently taking into consideration country, age, language, and mode of data collection (online or face-to-face) differences. Two sequences of successive multiple group confirmatory factor analyses were used to assess the psychometric properties of the OFQ, between: (a) emergent adults from the United States of America (*N* = 482, M_age_ = 25.23, *SD* = 2.746) and Australia (*N* = 168, M_age_ = 23.55, *SD* = 3.37) and (b) adolescents from Greece (*N* = 1579, M_age_ = 16.12, *SD* = 0.849) and Cyprus (*N* = 1372, M_age_ = 15.54, *SD* = 0.656). Configural and partial metric invariance were confirmed between the United States and Australian samples. For the Greek and Cypriot samples, results indicated full configural and metric invariance. These results provide initial information to researchers and clinicians of the extent to which the OFQ maintains its consistency when used across cultures and invite for further cross-cultural studies in the field. Implications, as well as limitations, are discussed.

## Introduction

In order to explain human motivation, researchers have offered several diverse explanations, including those that foreground biological, psychological and holistic factors (Alexandraki et al., 2018; Brunstein, 2018; Scerri et al., in press). Within the broader literature, two distinct forms of motivation have frequently been proposed. These are “implicit motives,” which target affective satisfaction, and “explicit motives,” which mostly relate to cognitive drives (Brunstein, 2018). Maslow (1943) proposed that motivation and behaviors are best explained by a hierarchy of needs, with survival needs (e.g., food and water) preceding higher tier motives. In line with Maslow’s approach, several forms of “implicit” motives (e.g., gratification) and/or “explicit” motives (e.g., drive for self-awareness and personal growth) might well be considered as higher-tier needs. However, a hierarchical conceptualization of motivation may not sufficiently explain why some people could abdicate instinctual urges including food and water in order to accomplish a task which gratifies higher-tier needs (Nakamura and Csikszentmihalyi, 2014; Whippman, 2017). Csikszentmihalyi (1975) highlighted that individuals who intensely engage in forms of play, hobbies and other absorbing activities might often prioritize their enjoyment of these activities over their biological needs. Such engagement may be due to the individual experiencing a state of “*Flow”* (Csikszentmihalyi, 1990; Nakamura and Csikszentmihalyi, 2014; Hu et al., in press). Flow has been theorized to include: (1) intense concentration, (2) a merging of action and awareness, (3) a loss of self-concern, (4) a control over one’s capabilities to engage in the activity, (5) an altered sense of time, (6) an engagement with task goals, (7) the receiving of immediate feedback, (8) a deep immersion in the activity, and (9) an experience of intrinsic reward – also known as an *autoletic* experience. When a person experiences these components concurrently, they are perceived to be operating at an “optimal” psychological state, defined as the state of Flow (Csikszentmihalyi, 1990; Nakamura and Csikszentmihalyi, 2014; Hu et al., in press).

The type of environment that best accommodates Flow is one where a challenging demand closely matches high self-efficacy and skill. In contrast, in cases where self-efficacy and skill exceed the challenges of the task, and where the challenge exceeds the individual’s skills, the person inclines to experience boredom and anxiety, respectively (Nakamura and Csikszentmihalyi, 2014). However, when in a state of Flow, one becomes completely immersed in an activity, leaving him/her with no residual mental energy. Despite the latter, Flow is typically viewed as a rewarding and positive force, enabling the individual to productively focus on goal-directed activities. For this reason, Flow has traditionally been examined in the context of positive psychology, involving areas of employment (Csikszentmihalyi and LeFevre, 1989; Salanova et al., 2006), educational learning (Shin, 2006), leisurely activities (Csikszentmihalyi and Bennett, 1971), creative processes (Csikszentmihalyi, 1990), and sports (Jackson et al., 1998).

Not surprisingly, the broad scope of activities that can elicit a state of Flow have prompted interest in understanding the potential biological basis of Flow. For instance, using Functional Magnetic Resonance Imaging (fMRI) scanning, Klasen et al. (2012) identified the involvement of the neocerebellum and the left primary and secondary somatosensory cortex with Flow experience occurring during game play. Regarding the particular associations between neurophysiological functioning and specific components of Flow, the midbrain reward structures (i.e., caudate nucleus, nucleus accumbens, putamen, thalamus, cerebellum, motor and premotor areas and the super parietal cortex) were associated with the perception of balance between ability and challenge. Furthermore, concentration and focus, also related to the state of Flow, were linked with increased activation in the cerebellum, visual systems, precuneus and premotor areas. In that line, clear task goals (which are considered precipitating and perpetuating factors of Flow), were shown to elicit increased activation of the bilateral intraparietal sulcus and fusiform face area. Finally, Klasen et al. (2012) revealed that a sense of control over the activity, as a Flow prerequisite, was characterized by increased activation of cortical regions (i.e., cerebellar, thalamic motor, and visual networks). Interestingly, the examination of certain neurobiological aspects of Flow appears to have been developing simultaneously with the investigation of its applicability online.

### Online Flow

The internet has been massively adopted to facilitate daily functioning (including survival needs; Dang et al., 2018) and across a variety of domains (e.g., employment, entertainment, communication and health; Anderson et al., 2017). Indicatively, the internet holds a pivotal role in the delivery of health services, as well as the accommodation of crucial physiological needs and even food choices (Zhang and Ho, 2017; Dang et al., 2018). These have inevitably triggered questions considering whether the online environment could endorse a state of Flow (Stavropoulos et al., 2013, 2018c; Anderson et al., 2017). Pioneering this area of research, Chen et al. (1999) postulated that feelings of enjoyment elicited by Internet use can be explained by Flow occurring whilst online. Results from their study suggested that the Internet is indeed an environment that facilitates Flow. Specifically, retrieving information, writing and reading emails, playing online games and online discourse were found to be Flow-inducing activities. Since then, online Flow has been explored in other, more specific online contexts. For instance, researchers have suggested that Flow can have a positive effect on learning outcomes in online educational applications (Shin, 2006). Following that line, the construct of online Flow has been often linked with *gamification* – which is the use of game mechanics (e.g., collecting points, leveling up) to engage users and increase action (Kapp, 2012). Gamification (when applied in an online educational context) has been supported to increase student engagement and motivation (Barata et al., 2013; Buckley and Doyle, 2016), and by extension, student academic performance. Such findings prompted, O’Donovan et al. (2013) to compare student grades from the same Computer Games Development course (at the tertiary level of education) and an updated course, which implemented gamification features. The results showed that student performance in the course with the improved game features had statistically significantly increased, compared to the academic performance of the students in the original non-gamified course. In that line, it has been additionally advocated that online Flow could be valuably utilized in e-health applications (Pañella, 2011). This, among others, could enhance user-experience and improve engagement in internet-based psychotherapies (Bederson et al., 2004; Fleming et al., 2017; Zhang and Ho, 2017; Zhang et al., 2018a).

Although such potentially positive effects have been widely supported, some scholars have recently identified online Flow as a risk factor for the overuse of internet applications and even reduced need for sleep (Stavropoulos et al., 2013, 2017a; Marino and Spada, 2017; Zhang M.W. et al., 2017; Peeters et al., 2018; Zhang et al., 2018c). Considering online gaming in particular, Flow related gamification aspects might lead to cognitive biases, which result in disproportionate attention to the game and could thus compromise engagement to competing educational stimuli (due to difficulties in disengaging from Internet use; Cristea et al., 2016; Hyrynsalmi et al., 2017; Zhang et al., 2018d). This is further reinforced by literature supporting that: (a) attention bias can act as an important mediator between individual characteristics and internet abuse behaviors (Zhang et al., 2018b); (b) gamification and cognitive bias should be therapeutically addressed in psychological treatment for addiction (Zhang et al., 2018a) and; (c) Immersive virtual reality therapy involving gamification features could be effective in treating excessive gaming (Zhang and Ho, 2017). Overall, it is assumed that gamification processes could escalate into a form of addiction (Zhang et al., 2018b). The latter has attracted significant attention particularly after the introduction of *Internet Gaming Disorder* (IGD), as a disorder requiring further study (American Psychiatric Association [APA], 2013; Hu et al., in press).

### Internet Gaming Disorder (IGD)

The excessive use of video-games (hereafter, games) leading to detrimental physical and psychosocial consequences has invited wellbeing concerns (Pontes and Griffiths, 2015; Stavropoulos and Hu, 2018). Psychologists and psychiatrists often define this “persistent and recurrent” pattern of gaming as potentially indicative of IGD, a syndrome described in the fifth edition of the *Diagnostic Statistical Manual of Mental Disorders* (DSM-5; American Psychiatric Association [APA], 2013, p. 795; Stavropoulos et al., 2018b). Recently, the World Health Organization (WHO) also recognized the detriment that excessive gaming can have on people by accepting and acknowledging *Gaming Disorder* (not necessarily via the Internet) as a clinical condition in their *International Classification of Disorders – 11* (ICD-11; World Health Organization [WHO], 2018). The attention given to maladaptive-abusive gaming behaviors, triggered a global endeavor to better understand this phenomenon (Petry et al., 2014; Adams et al., 2018; Burleigh et al., 2018; Liew et al., 2018).

Accordingly, several associated factors of IGD have been explored, including characteristics of the individual (e.g., age, self-esteem), the real-world context (e.g., family cohesion and occupational factors), and the gaming context-action (Beard and Wickham, 2016; Stavropoulos et al., 2016b). For instance, considering risk factors related to the gamer, in a systematic review, Kuss and Griffiths (2012) noted that features, such as loneliness, avoidant tendencies, neuroticism, and low self-esteem are linked to IGD. Additionally, numerous other psychopathological symptoms including depression, alcohol misuse, social anxiety, hostility/aggression, obsessive–compulsive symptoms, and attention deficit hyperactivity disorder manifestations have been associated to higher IGD risk (as factors related to the gamer; Ko et al., 2012; Ho et al., 2014; Stavropoulos et al., 2016a, 2019). However, the direction of causality considering such associations has been debated (i.e., IGD as the cause or result of comorbid psychopathological symptoms; Ciarrochi et al., 2016; Stavropoulos et al., 2019). Nevertheless, some scholars postulate that excessive gaming, similar to the excessive use of other recreational internet applications (i.e., social networking sites), may often act as a mechanism of addressing the discomfort related to real-world issues/problems, including pre-existing psychopathological symptoms (Ko et al., 2012; Plante et al., 2018). This hypothesis has been reinforced by findings suggesting that maladaptive coping styles (i.e., avoidance) partially explain the relationship between IGD and poorer mental health (Loton et al., 2016; McNicol and Thorsteinsson, 2017).

Considering contextual factors, Stavropoulos et al. (2017a) found that classrooms with higher percentages of Massively Multiplayer Online Role Playing Games (MMORPGs) paradoxically reduced the risk of internet abuse behaviors. Specifically, they suggested that MMORPG-related social connectedness could advance in-classroom relationships, reducing isolation and ultimately, risk of addiction (Stavropoulos et al., 2017a). Other significant contextual factors emphasize parenting and the family context. More specifically, poor family cohesion, high family conflict, and cold, unsupportive and uninvolved parenting practices have been reported as risk factors for the development and maintenance of IGD (Yen et al., 2007; Li et al., 2014; Adams et al., 2018).

### Online Flow and IGD

Considering gaming activity related risk factors, the psychological state of Flow online has been repeatedly shown to play an important role in the development and persistence of behaviors associated with IGD (Chou and Ting, 2003; Stavropoulos et al., 2013; Hu et al., in press). At this point it should be reiterated, that despite the noted benefits of Flow in online contexts, some researchers have repeatedly suggested that Flow may be implicated in youth and adult IGD behaviors, as well as excessive internet use in general (Chou and Ting, 2003; Wan and Chiou, 2006; Stavropoulos et al., 2013, 2018c). For instance, Stavropoulos et al. (2013) provided evidence that Flow was significantly associated with Internet abuse and this relationship was moderated by (tele-)presence, which reflects the perceptual state of being in an environment while the physical body of the user is in another (Witmer and Singer, 1998; Stavropoulos et al., 2018c). Later on, Stavropoulos et al. (2018c) used longitudinal data to assess variations in the risk effect of online Flow considering the development of Internet Addiction behaviors in adolescence and concluded that this remains significant despite developmental changes occurring between 16 and 18 years of age. Similarly, Lee et al. (2012) showed that the gamers experiencing increased frequency of online Flow, also reported increased use of Internet games. In a more recent study, Hu et al. (in press) supported that online Flow could partially explain the higher risk of online games involving socializing aspects for IGD symptoms. In line with these results, the positive feelings experienced during the Flow state have been suggested to be a critical risk factor for excessive online gaming in several relevant literature reviews (Kuss and Griffiths, 2012; Anderson et al., 2017). Conclusively, the positive feelings from the initial Flow experience could, at a later stage, manifest into a more compulsive pursuit for online gratification, causing people to behave addictively (Lee et al., 2012; Stavropoulos et al., 2018c; Hu et al., in press).

Indeed, Flow and addiction seem to present significant conceptual overlaps, which prompt reasonable comparisons (Stavropoulos et al., 2018c). For example, both addiction and Flow involve preoccupation with an engaging stimulus, signifying greater time spent in an activity, due to a combined altered sense of time and mood modification drives, which may generate a sense of loss of control (Chou and Ting, 2003; Tran et al., 2017a,b). Furthermore, while it is commonly accepted that tolerance and withdrawal symptoms are core components in addiction (Griffiths, 2005), Hu et al. (in press) as well as Stavropoulos et al. (2018c) hypothesized that online Flow may also have a dosage effect. Specifically these studies implied that gamers need to participate more frequently and intensely in gaming to satisfy their craving for Flow (Stavropoulos et al., 2018c; Hu et al., in press). This may explain why gamers, who play game genres that are more inducive of online Flow (e.g., MMORPGs; Johnson et al., 2012), are at a greater risk of addiction compared to gamers playing game genres that are less inducive of online Flow (Lee et al., 2007; Lemmens and Hendriks, 2016). Nevertheless, the fine line between Flow and addiction is demarcated by their impact on daily functioning (Tran et al., 2017a,b). One could argue that for most people, Flow is a healthy state with proper functional engagement to an activity, while addiction is an unhealthy state caused by excessive involvement in an activity, leading to a loss of control over the activity (while Flow does not; Tran et al., 2017a,b). In this context, Flow usually enhances, rather than impairs daily functioning (Salanova et al., 2006), while addiction compromises it, especially through withdrawal effects (Lai et al., 2013; Tran et al., 2017a,b).

Given the considerable influence that online Flow is posited to have on reinforcing adaptive, as well as problematic Internet use and Internet gaming behaviors specifically, greater research is needed to further understand its potential contributions. In order to do so, however, and given the global nature of the research conducted in this field, it is vital to examine the psychometric properties of commonly employed instruments that purport to measure online Flow across different cultural groups. Thus, the present study focuses on the measurement consistency of online Flow across different national populations by cross-culturally assessing the psychometric properties of the widely used Online Flow Questionnaire (OFQ; Chen et al., 1999). This, among other benefits (i.e., general assessment accuracy and comparability), could pave a more reliable and valid way for future studies examining the online Flow and IGD link.

### Online Flow Questionnaire (OFQ)

A noteworthy and a widely used instrument of assessing online Flow is the OFQ (Chen et al., 1999). This entails five binary (0 = No, 1 = Yes) coded items examining online Flow features (e.g., “Have you ever experienced the feeling of ‘positive challenge’ during your Web navigation/online?”). Endorsed items are summed to a range from 0 to 5, with 0 indicating minimum and 5 indicating a maximum rate of online Flow experiences. Researchers who have previously employed the OFQ reported a single factor structure and adequate Kuder-Richardson reliability coefficients (0.71; Stavropoulos et al., 2013; 0.70; Stavropoulos et al., 2018c). However, such evidence, although useful considering internal reliability perspectives, is somewhat rudimentary in that it fails to provide critical psychometric information regarding the use of the scale for comparisons across groups (e.g., cultural groups, genders, types of gamers). Further exploration of these facets of measurement would advance knowledge of online Flow by confirming the consistency of the one-factor structure, item loadings, and score meanings (regarding the magnitude of online Flow experienced) across different cultural groups (Stavropoulos et al., 2018a). Conclusively, the need to assess the psychometric equivalency of the OFQ across different populations of gamers is crucial provided: (a) the global nature of the Internet, and (b) the pivotal contribution of online Flow to both adaptive (i.e., e-health applications employing gamification) and maladaptive Internet use, such as IGD. At this point it should be noted that past studies have indicated that diverse age groups (i.e., adolescent vs. adults; Kwiatkowska and Rogoza, 2017), languages of delivery (i.e., English vs. Greek; Zavala-Rojas and Saris, 2017) and modes of data collection (i.e., online vs. face to face; Zhang X. et al., 2017) can also interfere with the pattern of addressing items of psychological scales, thus distorting their psychometric properties. Therefore, such disparities should be controlled (for psychometric variations related to cultural differences to be adequately and specifically studied; Gomez and Stavropoulos, 2018).

### The Role of Culture

Social structures across countries have been envisaged to lie on a continuum ranging from more individualistic to more collectivistic (Hofstede, 1984; Triandis, 2018). The individualism–collectivism dimension refers to the extent to which decision-making processes are more or less influenced by individual interests, goals and/or aspirations, compared to those of the group(s) to which the individual belongs (e.g., family, peers, community; Hofstede, 1984; Triandis, 2018). In that line, countries such as Greece and Cyprus appear to align closer to collectivistic tendencies, whereas countries like Australia and the United States, appear to reflect a more individualistic societal structure (Hofstede, 1984; Triandis, 2018). The individualism–collectivism aspect has been suggested to be complemented by the dimension of “horizontality–verticality,” which describes the extent of concurrent equality–inequality (i.e., inequality in social benefits and access to services) experienced within a culture (Triandis, 2018). In this context, the social and state functions in the United States have been assumed to be more “vertically” individualistic than those in Australia (Stavropoulos et al., 2018a). Similarly, Greece has been defined as being more horizontally collectivistic than Cyprus (likely due to Cyprus’ exposure to the British Commonwealth; Stavropoulos et al., 2018a). Such differences in cultural features have been shown to associate with online behaviors in general and gaming involvement patterns in particular (Lee and Wohn, 2012; Xu-Priour et al., 2014; Hong, 2015) and to impact the psychometric equivalence of measures (Stavropoulos et al., 2017b). For instance, findings from a measurement invariance (MI) study of the Internet Gaming Disorder Scale – Short Form 9 between the United States, the United Kingdom, and India revealed that despite cross-country consensus of a one-factor structure of the instrument, there were cross-country differences on the metric and scalar level of analyses (Pontes et al., 2017). As online Flow is a globally applied construct, differences and similarities of the related measurement properties across countries are particularly important (lack of awareness of such differences could confound the interpretation of research findings).

### Measurement Invariance

One method to assess the cross-cultural psychometric equivalency of the instrument is to conduct MI analysis. MI analysis enables researchers and clinicians to be able to compare results or scores from an instrument across different groups (e.g., cultural groups) with the assurance that deviations in observed scores are due to unique differences of individuals assessed, rather than measurement or instrument artifacts. In brief, MI concerns three levels of analysis with each level progressively becoming more stringent. The three levels are (i) configural invariance, (ii) metric invariance, and (iii) scalar invariance (Milfont and Fischer, 2010). Configural invariance examines the *factor structure* of an instrument across different groups. Metric invariance examines whether the *factor loadings* of the items vary across different groups or the relative strength of each indicator on the latent factor. Scalar invariance is concerned with the degree to which participants from different groups conceptualize and respond to the items on the instrument in the same way (i.e., same numbers indicating the same level of intensity for the item assessed). Scalar invariance can only be meaningfully analyzed in instruments and items that entail more than two response options (i.e., binary items do not provide an adequate range to assess intercept and/or threshold differences). At any one of the three levels, should the analysis reveal unsatisfactory full invariance, then a partial invariance can be conducted by inspecting the modification indices and successively relaxing the appropriate items until partial invariance is achieved (Reise et al., 1993). These three tests are necessary to be met for cross-group comparisons. There are, however, other optional tests (e.g., error variance, factor mean invariance, factor variance invariance, factor covariance invariance) that differ in scope, purpose and restrictiveness and have been often considered as un-necessary (see Steenkamp and Baumgartner, 1998; Milfont and Fischer, 2010; Brown and Moore, 2012). In that line, to ascertain that differences in the psychometric properties assessed are exclusively related to the cultural variations targeted (i.e., culture/country), groups compared need to be matched in regards to other potentially confounding features, such as the mode of data collection (i.e., face to face and/or online; Zhang X. et al., 2017), the language of the scale (i.e., Greek vs. English; Zavala-Rojas and Saris, 2017) and age ranges (i.e., adolescents vs. adults; Kwiatkowska and Rogoza, 2017).

### The Present Study

Given the universal nature of the Flow phenomenon, it is worthwhile (if not imperative) exploring the psychometric consistency of online Flow measurements in diverse populations. In doing so, the results will be more representative of the modern human experience (Rad et al., 2018). To the best of the authors’ knowledge there have been no previous studies addressing this topic. To address this gap in the literature, the present work will aim to examine MI of the OFQ across cultures/countries, by conducting two different studies which implement a sequence of CFA models across two pairs of samples, matched regarding their age ranges (adolescents and adults), the language of delivery of the OFQ (Greek and English) and the mode of data collection (face to face and online), such that the differences assessed would associate exclusively with the country/culture of origin. These pairs of countries have been carefully selected to culturally reflect similar levels of individualism–collectivism, concurrently with variations considering horizontality–verticality (Triandis, 2018). Findings are expected to provide information about the comparability of OFQ scores across diverse cultures (i.e., OFQ items that may require a more careful interpretation are expected to be revealed) and therefore, to pave the way for broader cross-cultural studies and greater adoption of the OFQ for reliable comparisons of online behaviors worldwide. In particular:

***Study 1*** involves MI examinations, considering a pair of emergent adult samples of the OFQ, delivered in English, collected online and originated from the United States and Australia.

***Study 2*** involves MI examinations, considering a pair of adolescent samples of the OFQ delivered in Greek, collected face to face and originating from Greece and Cyprus.

## Materials and Methods

### Participants

#### Study 1

Participants were emergent adults Internet gamers (*N* = 650) were recruited online from the United States from Amazon Mechanical Turk (*N* = 482, Age_Min_ = 18, Age_Max_ = 29, *M* = 25.23, *SD* = 2.746; 57.1% Males, Min_Internet Game Use Duration in years_ = 1, Max_Internet Game Use Duration in years_ = 30 M_Internet Game Use Duration in years_ = 10.95, *SD* = 5.32, Min_Internet Game Use Duration per week in hours_ = 0, Max_Internet Game Use Duration per week in hours_ = 100, M_Internet Game Use Duration per week in hours_ = 13.10, *SD* = 11.50) and Australia (*N* = 168, Age_Min_ = 18, Age_Max_ = 29, *M* = 23.55, *SD* = 3.37; 78% Males; Min_Internet Game Use Duration in years_ = 6, Max_Internet Game Use Duration in years_ = 22 M_Internet Game Use Duration in years_ = 14.19, *SD* = 3.68, Min_Internet Game Use Duration per week in hours_ = 1, Max_Internet Game Use Duration per week in hours_ = 50, M_Internet Game Use Duration per week in hours_ = 8.64, *SD* = 8.06). The estimated maximum sampling error for the United States sample of 482 is ±4.46% at the 95% level of confidence. With a sample size of 168 for the Australia sample, the estimated maximum sampling error is ±7.56% at the 95% level of confidence. As for the total sample size, the estimated maximum sampling error is ±3.84% at the 95% level of confidence. Participation was voluntary, with no incentives offered.

#### Study 2

In the second study, the total paper–pencil survey sample (*N* = 2951) comprised high school students from Greece (*N* = 1579, Min_age_ = 15, Max_age_ = 20, M_age_ = 16.12, *SD* = 0.849, Min_Internet Use Duration in years_ = 1, Max_Internet Use Duration in years_ = 6 M_Internet Use Duration in years_ = 3.26, *SD* = 1.24, Min_Internet Use Duration of the preferred Internet application per weekday day in hours_ = 1, Max_Internet Use Duration of the preferred Internet application per weekday day in hours_ = 3M_Internet Use Duration of the preferred Internet application per weekday day in hours_ = 2.04, *SD* = 0.77; Min_Internet Use Duration of the preferred Internet application per weekend day in hours_ = 1, Max_Internet Use Duration of the preferred Internet per weekend day in hours_ = 3 M_Internet Use Duration of the preferred Internet application per weekend day in hours_ = 2.20, *SD* = 0.79) and Cyprus (*N* = 1372, Min_age_ = 14, Max_age_ = 19, M_age_ = 15.54, *SD* = 0.656, Min_Internet Use Duration in years_ = 1, Max_Internet Use Duration in years_ = 6 M_Internet Use Duration in years_ = 4.23, *SD* = 0.88, Min_Internet Use Duration of the preferred Internet application per weekday day in hours_ = 1, Max_Internet Use Duration of the preferred Internet application per weekday day in hours_ = 3 M_Internet Use Duration of the preferred Internet application per weekday day in hours_ = 2.09, *SD* = 0.77; Min_Internet Use Duration of the preferred Internet application per weekend day in hours_ = 1, Max_Internet Use Duration of the preferred Internet application per weekend day in hours_ = 3 M_Internet Use Duration of the preferred Internet application per weekend day in hours_ = 2.35, *SD* = 0.75). The estimated maximum sampling error for the Greek sample is 2.32% at the 95% level of confidence. Response and parental consent rates reached 95%. The Greek data was collected in class during 2011. The Cypriot data was collected identically in 2012. The estimated maximum sampling error for the Cypriot data is 2.70% at the 95% level of confidence. Response and parent consent rates were similarly obtained for over 95% of the sample. Participation was voluntary, with no incentives offered.

### Measures: Studies 1 and 2

#### Online Flow Questionnaire

To assess online Flow, Study 1 and Study 2 employed the OFQ delivered in English (see Appendix A) and Greek, respectively (Chen et al., 1999). Considering Study 2, the adapted Greek OFQ (see Appendix B), that was produced after bi-directional translations from bi-lingual translators, was used (Stavropoulos et al., 2013). The OFQ consists of five pairs of self-report questions relating to online Flow experiences (i.e., have you experienced the feeling of positive challenge when playing your preferred online game (0 = No, 1 = Yes). Participants are then asked to name the application in which this occurred. A final score is computed by summing the scores for the first question in each of pair of items. The scores can range from 0 to 5 representing minimal and maximal experience of online Flow. Based on literature recommendations (Revelle and Zinbarg, 2009), the internal reliability of the present questionnaire was calculated using the McDonald’s (1999) omega (Ω) reliability index. While Cronbach’s alpha assumes that item factor loadings are all equal, Omega allows factor loadings to vary (which was the case in the present samples; Revelle and Zinbarg, 2009). Values of the omega reliability coefficient can be considered as acceptable in line with the cut-off points suggested for the Cronbach’s alpha coefficient. Specifically, values above 0.70 tend to be considered acceptable and over 0.80 tend to be preferred (Viladrich et al., 2017). Overall, Omega values have been satisfactory for the American sample (Ω = 0.87), the Australian sample (Ω = 0.98), the Greek sample (Ω = 0.74), and the Cypriot sample (Ω = 0.75). At this point it needs to be noted that the reliability values for the current study align with those of previous studies referring to similar national populations (Stavropoulos et al., 2013; Hu et al., in press).

### Procedure

#### Study 1

Data used in the present study are derived from a larger study on risk and resilience factors associated with IGD in emergent adulthood. Online data collection surveys were developed in tandem for the American and Australian sample. Data collection, as well as its use for prospective research purposes (such as the present study), was approved by the Human Research Ethics Committees from Federation University in Ballarat, Victoria and Palo Alto University in Palo Alto, California. Therefore, according to relevant institutional and national guidelines and regulations, full review and approval for the present MI study was not required. Participants were recruited online. Eligible individuals (i.e., adults gamers, permanent residents, or citizens of the countries involved), who were interested in participating were invited to the study via a SurveyMonkey link (for the Australians) or an Amazon Mechanical Turk link (for the Americans). For both samples, the survey was advertised across numerous gaming websites and forums. The URL link to the study directed prospective participants to the Plain Language Information Statement (PLIS). The PLIS explicitly indicated that participation was entirely voluntary and that participants were free to withdraw from the study at any time before completion. Furthermore, participants were informed that any discontinuation, at any point, required no explanation and was without any penalties. Participation, completion and submission of the questionnaire was only possible after participants provided their informed consent to partake in the study. Participants who chose to participate in the study were directed to a question regarding informed consent. If participants clicked “yes” to providing informed consent, they were then guided to the questionnaire battery. Participants who clicked “no” were directed to the exit page and thanked for their time.

The preference for online data collection over traditional paper-and-pencil methods was guided by relevant literature. Not only are online data collection methods more cost-effective, but they can access hard to reach groups that were relevant to the present study (i.e., gamers; Griffiths, 2010). Additionally, online data collection can acquire data from participants of diverse backgrounds, and thus, is considered appropriate for psychological research (Casler et al., 2013; Chandler and Shapiro, 2016). Scholars have also shown that online data collection and paper-and-pencil methods are generally equivalent (Pettit, 2002; Weigold et al., 2013).

#### Study 2

Data collection for the Greek and Cypriot study, as well as its use for prospective research purposes (such as the present study), received approvals from the ethical committees of the University of Athens and the Cypriot Youth Organization, respectively. Similar to *Study 1*, full review and approval for the present MI study was not required according to relevant institutional and national guidelines and regulations. Data collection was identical for the Greek and Cypriot sample. Written permission to conduct the study came from: (i) The Ministries of Education; (ii) The Teachers’ Council; and (iii) written informed consent was obtained from parents and/or guardian of all non-adult participants. In both countries, the ratios of schools and students were identified based on (i) location and (ii) the type of school (i.e., academic vs. vocational track schools). Based on these stratifications, participants were then selected randomly by lottery. There were no exclusion criteria because the aim of the studies was to collect inclusive and representative samples from the two countries. Data was collected in class. In both countries, this process took no more than two school hours.

### Statistical Analyses: Studies 1 and 2

Analyses were conducted in Mplus, version 7 (Muthén and Muthén, 2012). The sequence of models tested followed previous MI studies (e.g., Pontes et al., 2017) and MI literature (Milfont and Fischer, 2010). Specifically, sequential analyses were conducted whereby the level of invariance (i.e., configural and metric) was progressively tested for both studies. Given the binary structure of responses to the OFQ, scalar invariance was not tested. When full metric invariance is not supported, the source of the non-invariance can be identified by progressively releasing the factor loadings with the highest modification index until partial metric invariance is achieved. The Weighted Least Squares Means and Variance (WLSMV)^1^ χ^2^ difference values and the corresponding differences in the *df* values (Muthén and Muthén, 2012) were used to examine model fit differences. Given the sample sizes and the associated sensitivity of χ^2^, the significance level was set at 0.01 to allow for more rigorous control of Type 1 errors (Gomez and Stavropoulos, 2018).

In both studies, the Tucker-Lewis Index (TLI), Comparative Fit Index (CFI), and Root Mean Square Error of Approximation (RMSEA) were used to estimate the model’s goodness of fit. These indices were chosen because they are robust measures in the presence of complex models (Kenny, 2014). As a result, and not surprisingly, these measures have been widely used in previous MI studies (e.g., Gomez and Suhaimi, 2015; Pontes et al., 2017; Stavropoulos et al., 2017b).

### Data Screening and Preparation

#### Study 1

Missing data from the United States and Australian samples were first assessed using Little’s Missing Completely at Random (MCAR) test to determine the pattern of missing data. Results indicated that the data was missing completely at random (χ^2^ = 42.36, *df* = 26, *p* = 0.023). Thus, to avoid reducing sample power due to listwise deletion (Schlomer et al., 2010), missing values were replaced using Full Information Maximum Likelihood (FIML). This is a default missing values procedure in *Mplus7* (Muthén and Muthén, 2012). It does not impute missing values but rather, gives an estimation of what those missing values could be (Schlomer et al., 2010). Additionally, screening for multivariate outliers was performed at the item-level by plotting the outlier log-likelihood provided by Mplus7. This yielded a visual representation of the multivariate outliers. No multivariate outliers were found.

#### Study 2

The same missing values related process was adopted for Study 2 for the Greek and Cypriot data. Little’s MCAR test also indicated here that the data was missing completely at random (χ^2^ = 68.83, *df* = 48, *p* = 0.026). Accordingly, FIML was also applied to replace missing values, and no multivariate outliers were revealed.

## Results

### Confirmatory Factor Analysis and Measurement Invariance Outcomes

#### Study 1

First, analyses examined separately the OFQ reliability and model fit across the two populations and then the MI models. Table 1 presents the descriptive statistics, intercorrelations and the Kuder-Richardson and Omega (ω) reliability coefficients for the OFQ across groups (United States–Australia). The scale showed acceptable reliability indices between nationalities.

**Table 1:** Descriptive statistics and reliability coefficients for the Australian and United States sample.

	Australian sample (*n* = 168)					United States sample (*n* = 482)				
	*M*	*SD*	MIC	K-R	Ω	*M*	*SD*	MIC	K-R	Ω
Flow	4.45	1.34	0.682	0.91	0.98	3.86	1.32	0.317	0.67	0.83

MIC, mean inter-item correlations; Ω, McDonald’s omega reliability (based on standardized values); K-R, Kuder-Richardson reliability (based on standardized values).

**Figure 1:**
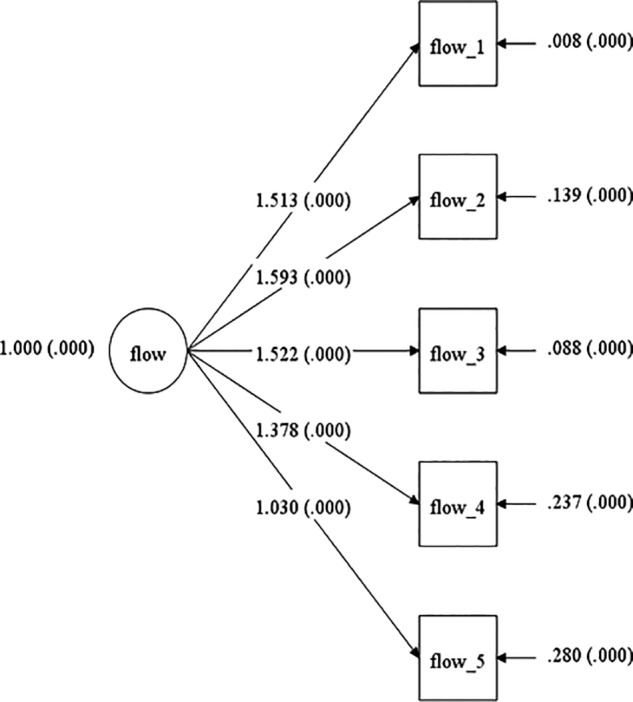
Model for the Australian sample.

**Figure 2:**
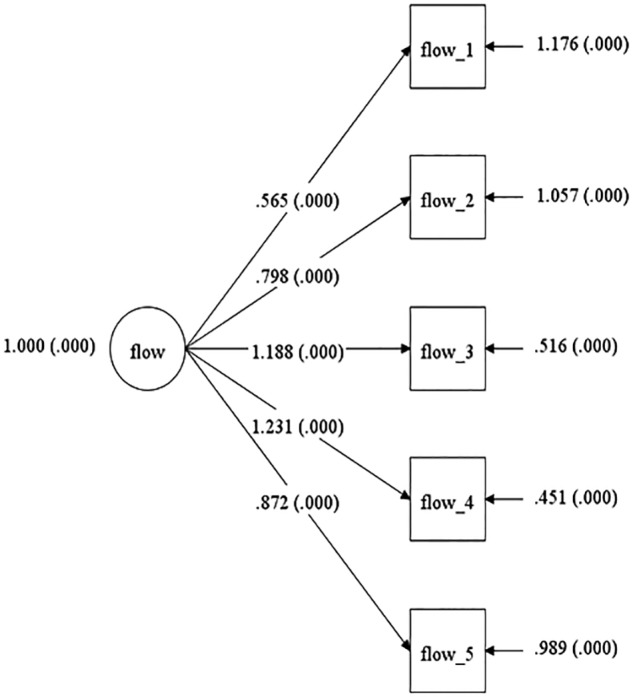
Model for the United States sample.

The model CFA demonstrated good fit, even on the basis of absolute fit indices for the Australian sample (χ^2^ = 6.473, *df* = 5, *p* = 0.263, CFI = 0.999, TLI = 0.999, RMSEA = 0.048; see Figure 1). As for the United States sample the chi-square value was significant (χ^2^ = 15.487, *df* = 5, *p* = 0.0085, CFI = 0.973, TLI = 0.946, RMSEA = 0.068; see Figure 2), indicating a lack of absolute fit for the one-factor structure of the OFQ. However, due to the sensitivity of the chi-square test to the sample size, the degree of correlations in the model (Kenny, 2014), and in line with past MI studies (Gomez and Stavropoulos, 2018), incremental measures of fit guided the interpretation of the model. Following benchmarks recommended in the literature the CFI^2^, TLI^3^, and RMSEA^4^ coefficients were deemed indicative of a good incremental model fit. In addition, all standardized factor loadings for the model across both populations, were statistically significant (i.e., *p* < 0.01). Loadings were above 0.89 and 0.46 for the Australian and United States sample, respectively. The unconstrained multi group (i.e., configural invariance) model (M1) was estimated with an acceptable incremental fit (χ^2^ = 21.695, *df* = 10, *p* = 0.0167, CFI = 0.996, TLI = 0.992, RMSEA = 0.063). Finally, with factor loadings fixed and thresholds free, the Metric invariance model (M2) compared to the configural model resulted in a significant drop in fit indices (Δχ^2^ = 20.08, Δ*df* = 5, *p* = 0.0012). Inspecting the modification indices, indicated that the model fit would improve if the constraint for the factor loading of item 1 was relaxed between the two samples. Therefore, a final partial metric invariance model including this modification was calculated. The model fit for this partial metric invariance model did not differ significantly from that of the configural model (Δχ^2^ = 1.727 Δ*df* = 3, *p* = 0.6309). Table 2 depicts the model fit indices and results of the MI analyses. It should be noted, that these are based on a rather more conservative psychometric approach that infers invariance, based on absolute fit indices’ differences (Pontes et al., 2017). Nevertheless, according to more ‘lenient’ literature recommendations considering differences in incremental fit indices between successively nested models, a value of ΔCFI smaller than or equal to -0.01 (as presented here) indicates that the null hypothesis of invariance should not be rejected (Cheung and Rensvold, 2002) and therefore, full invariance could be inferred for the OFQ factor loadings without partial metric invariance needing to be conducted.

**Table 2:** OFQ MI across the Australian and United States sample.

	Model fit^1^						Model difference^2^		
	*χ*^2^	*df*	*p*	CFI	TLI	RMSEA	Δχ^2^	Δ*df*	*p*
M1: Configural: Loadings + intercepts free	21.695	10	0.0167	0.996	0.992	0.063	–	–	–
M2: Metric: Loadings fixed + intercepts free	43.393	15	0.0001	0.991	0.988	0.081	20.080	5	0.0012
M3: Partial invariance	18.321	13	0.1457	0.998	0.997	0.038	1.727	3	0.6309

^1^*Based on WLSMV. ^*2*^Based on ΔWLSMV.*.

#### Study 2

Similar to Study 1, OFQ internal reliability and model fit was calculated separately for the two samples. Table 3 presents the descriptive statistics, intercorrelations, and the Kuder-Richardson and Omega (ω) reliability for the OFQ across groups (Greece – Cyprus). The scale showed acceptable reliability indices between the two populations.

**Table 3:** Descriptive statistics and reliability coefficients for Greek and Cypriot sample.

	Greek sample (*n* = 1493)					Cypriot sample (*n* = 1301)				
	*M*	*SD*	MIC	K-R	Ω	*M*	*SD*	MIC	K-R	Ω
Flow	2.29	1.34	0.203	0.56	0.74	2.32	1.36	0.208	0.57	0.75

*MIC, mean inter-item correlations; Ω, McDonald’s omega reliability (based on standardized values); K-R, Kuder-Richardson reliability (based on standardized values)*.

**Figure 3:**
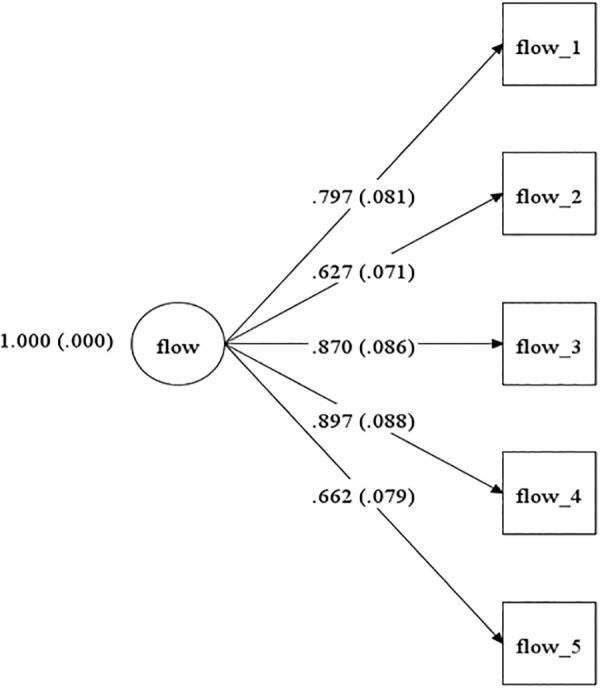
Model for the Greek sample.

**Figure 4:**
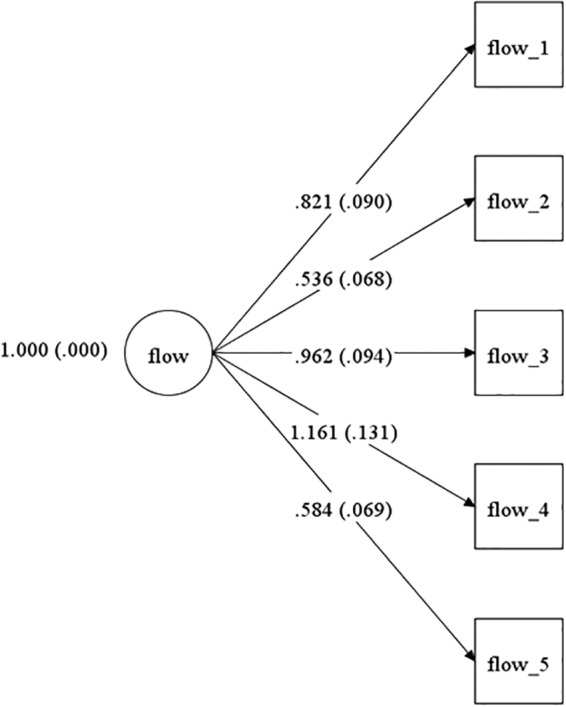
Model for the Cypriot sample.

The CFA had a good incremental fit for Greek (χ^2^ = 22.055, *df* = 5, *p* = 0.0005, CFI = 0.977, TLI = 0.954, RMSEA = 0.047; see Figure 3) and Cypriot participants (χ^2^ = 31.86, *df* = 5, *p* = 0.0000, CFI = 0.965, TLI = 0.929, RMSEA = 0.063; see Figure 4). All standardized factor loadings were statistically significant (i.e., *p* < 0.01). Loadings were above 0.53, and 0.47 for the Greek and Cypriot samples, respectively.

Following the CFA tests of model fit, the unconstrained multi group (i.e., configural invariance) model (M1) was computed with an acceptable fit as shown in Table 4 (χ^2^ = 53.826 *df* = 10, *p* = 0.0000, CFI = 0.971, TLI = 0.941, RMSEA = 0.055). As in Study 1 CFI, TLI and RMSEA were used as the as the main fit indices. Finally, with factor loadings fixed and thresholds free, the Metric invariance model (M2) compared to the configural invariance model did not result in a significant drop in fit indices (Δχ^2^ = 10.212, Δ*df* = 5, *p* = 0.0694).

**Table 4:** OFQ MI across Greek and Cypriot sample.

	Model fit^1^						Model difference^2^		
	*χ*^2^	*df*	*p*	CFI	TLI	RMSEA	Δχ^2^	Δ*df*	*p*
M1: Configural: Loadings + intercepts free	53.826	10	0.0000	0.971	0.941	0.055	–	–	–
M2: Metric: Loadings fixed + intercepts free	56.127	15	0.0000	0.973	0.963	0.043	10.212	5	0.0694

^1^*Based on WLSMV. ^*2*^Based on ΔWLSMV*.

## Discussion

The degree of Flow experienced online has been supported to play a substantial role in both adaptive as well as maladaptive uses of the Internet, and in particular the development and persistence of IGD behaviors, triggering global research interest (Chou and Ting, 2003; Wan and Chiou, 2006; Chiang et al., 2011; Hull et al., 2013; Stavropoulos et al., 2013, 2018c). Despite these, there has been a lack of cross-cultural MI examination, even regarding more commonly used scales assessing online Flow, such as the OFQ (Stavropoulos et al., 2018a). Lack of MI information across different cultural groups considering any scale, and in this case the OFQ, is problematic because studies cannot reliably infer differences involving diverse groups (as these could be confounded by discrepancies in the scale’s psychometric properties; Gomez and Stavropoulos, 2018). To contribute to this need, the present work applied MI analyses to evaluate the invariance of the psychometric properties of the OFQ, across two pairs of samples (*Study 1:* Australia and the United States; *Study 2:* Greece and Cyprus). These pairs were matched in terms of the language of delivery of the scale (*Study 1*: the OFQ in English; *Study 2*: the OFQ in Greek), the age ranges (*Study 1:* emergent adults; *Study 2:* adolescents) and the mode of the data collection (*Study 1:* online; *Study 2:* face to face), to concurrently control for factors influencing the analyses, other than that of the country of origin (Kwiatkowska and Rogoza, 2017; Zavala-Rojas and Saris, 2017; Zhang X. et al., 2017). In brief, and despite the results confirming the single factor structure of the OFQ across all four samples, group differences were revealed at the level of the metric invariance analysis (i.e., the strength of the items loading on the factor; Gomez and Stavropoulos, 2018). Specifically, while no differences were reported considering Greek and Cypriot adolescents (i.e., full metric invariance found in *Study 2*), for the American and Australian emergent adult Internet gamers, item one appeared to load significantly differently between the two samples. Results introduce caution regarding the establishment of the measurement equivalence of the OFQ across different cultures. This is particularly useful in light of the increasing worldwide research and clinical attention directed to online Flow (Stavropoulos et al., 2018b,c).

### Study 1: Australia and the United States

In the present study, participants from Australia and the United States were emergent adults Internet gamers (18–29), who were assessed online in English. As indicated by the model fit, both the Australian and the United States samples showed support for the single-factor structure model of the OFQ. In that line, results of the configural model revealed that both groups conceptualized the Flow construct similarly (i.e., in an invariant single construct way). Employing the configural model as the baseline, the subsequent metric invariance analysis revealed a significant drop in absolute fit indices. The origin of the non-invariance was narrowed down by inspecting the modification indices. Thus, when item one^5^ was relaxed in the partial metric model, the model’s fit converged significantly to that of the baseline (configural) model. Interestingly, lack of full metric invariance has been reported in other studies, referring to scales assessing psychological constructs highly associated to that of online Flow, such as IGD (Pontes et al., 2017; Stavropoulos et al., 2018a). For instance, examining the MI of a questionnaire measuring IGD behaviors, Stavropoulos et al. (2018a) found that there was a lack of full metric and scalar invariance between samples of Internet gamers deriving from Australia, the United Kingdom, and the United States, using the same United States and Australian samples.

The similarity in MI outcomes between the abovementioned study and the present study could be due to socio-cultural variations assumed to exist between the United States and Australia (Stavropoulos et al., 2018a). Specifically, it is likely that variations considering qualities of the cultural dimension of individualism could interfere with these results. Accordingly, instead of viewing individualism as a single construct, Singelis et al. (1995) emphasized the importance of disseminating individualism and collectivism into either horizontal or vertical variations. Vertical individualism refers to the idea that the individual is autonomous within a society that accepts inequality. Horizontal individualism differs (from its vertical counterpart) in the sense that society simultaneously emphasizes equality. From the collectivism perspective, vertical collectivism describes the individual as part of a collective, yet inequalities within the collective are accepted. Horizontal collectivism perceives the individual as part of the collective, while equality is concurrently emphasized. In this context, Australia has been envisaged to resemble a horizontally oriented individualistic society, while the United States has been supported to reflect a more vertically inclined individualistic society (Triandis, 2001; Pontes et al., 2017; Stavropoulos et al., 2018a). As a result, the difference in the way that society emphasizes (in)equality is thought to potentially contribute to the lack of full metric invariance, due to a response pattern that one uses to address the scale items. In fact, the effects of item bias are known to produce measurement non-invariance (Byrne and Watkins, 2003).

Specifically, items are assumed to be biased if they are interpreted differently across cultural groups. In this context, the disconnection and alienation described in item one of the OFQ is potentially perceived more broadly among United States gamers, due to potential cultural effects of vertical individualism (Stavropoulos et al., 2018a). Specifically, cultural effects could confound the way that the United States gamers report the level of disconnection (interfered with online Flow). Conversely, disconnection and alienation (related to the online Flow state), could be reported in a less diverse way by Australian gamers, who are typically perceived as more horizontally individualistic compared to their United States counterparts (Pontes et al., 2017; Stavropoulos et al., 2018a). Thus, given that item one reflects a rather holistic description of the online Flow construct, it is considered here that this is reported differently between United States and Australian emergent adult Internet gamers. As a result, the OFQ item one should be cautiously interpreted, if not reviewed and potentially carefully revised, when addressing comparisons between United States and Australian emergent adult Internet gamers.

### Study 2: Greece and Cyprus

Participants from Greece and Cyprus constituted representative adolescent samples, assessed face to face, within their classrooms, in Greek. The MI outcome for the Greek and Cypriot samples differed (to some extent) from that of *Study 1*. Here in *Study 2*, full invariance of the OFQ, at both the configural and the metric level was observed, indicating that there is consistency in the way that these two samples conceptualize the psychological construct of online Flow, as well as the way that all items associate to the latent construct. This aligns with literature suggesting consistency in the factor structure and the factor loadings related to the way that Greeks and Cypriots approach scales referring to psychological constructs, such as romantic attachment (Stavropoulos et al., 2017b). Interestingly though, these two Greek speaking populations, present differences considering the scalar invariance level, which reflects variations in the meaning of the same item scores (Stavropoulos et al., 2018a). Despite sharing similarities with Greece (i.e., language and religion), Cyprus was once an English colony and is currently a Commonwealth member, and thus, has been assumed to have been exposed to more individualistic tendencies (Terkourafi, 2007). This greater orientation toward individualism for the Cypriots, compared to the traditionally higher collectivism of the Greeks has been stipulated to interfere with the MI of psychological scales applied in these two countries (Stavropoulos et al., 2017b). Thus, affecting the scoring of the items (i.e., scalar invariance – same reported score meaning different levels of severity). However, given the binary nature of the OFQ items, the scalar invariance assessment was not feasible in the present study. Based on the present study’s findings, there is MI of both the factor structure and the item loadings across Greek and Cypriot adolescents. Ultimately, the implication of these results suggests that it is relatively safe to compare scores of the binary coded OFQ between matched populations from these two countries. However, cautiousness should be exhibited if future editions of the OFQ do not involve binary responses.

### Study 1 and Study 2

As previously mentioned, direct comparisons of the psychometric equivalence of the OFQ between the two groups of countries were not ideal in the present study. This is because different data collection methods and language of delivery of the OFQ were employed across different age ranges (Kwiatkowska and Rogoza, 2017; Zavala-Rojas and Saris, 2017; Zhang X. et al., 2017). Despite not being able to concurrently examine the psychometric equivalency of the OFQ across all four countries, it is postulated here that several factors are contributing to the different results observed across the two sets of countries.

Comparing the two studies, participant characteristics were dissimilar. In Study 1, participants were emerging adults (age: 18–29), who were self-described as gamers, coming from two multicultural societies, where populations are less consistent in the way they address psychological scales (Stavropoulos et al., 2017b). In Study 2, participants were representative groups of Greeks and Cypriots adolescents (age: 14–20), coming from non-multicultural countries, with similar ethnic background, and therefore possibly presenting with more consistent ways of addressing psychological scales (Gomez and Suhaimi, 2014). Given that American and Australian participants were older, their conceptualization of the OFQ, and in particular the description of Flow in item one, may have been more heterogeneous than the Greek and Cypriot participants (due to more lengthy and diverse experiences of online Flow). Moreover, since the younger population assessed in *Study 2* may have had fewer experiences of online Flow, they may have conceptualized the OFQ and specifically item one in a more homogenous way. Finally, the different modes in which the data collection was administered in *Study 1* and *Study 2*, may have played a role. Typically, data collected either online or by paper and pencil (but not both) is thought to be psychometrically similar (Pettit, 2002; Zhang et al., 2012). Reinforcing this idea, some MI studies have indicated that Internet-based methods present to be psychometrically different compared to paper and pencil methods, which could explain the weaker metric invariance revealed in *Study 1* compared to *Study 2* (Meade et al., 2007).

### Strengths and Limitations

The present work is a worthwhile contribution to cross-cultural psychology and e-psychology literature, with implications of the findings being valuable to researchers and clinicians. To the authors’ awareness, this is the first study worldwide that assesses the psychometric equivalence of the OFQ across different countries, age ranges, languages and data collection modes, while concurrently controlling for their potential confounding effects by implementing two MI analyses sequences. Establishing invariance and acknowledging the sources of non-invariance helps to give direction and guidance for future research and clinical assessments. Secondly, the present findings provide evidence that the comparability of the OFQ may be improved for emergent adult Internet gamers coming from multicultural societies if item one is addressed (cautiously interpreted or revised). As a result, this invites further research, especially involving Asian populations which tend to be significantly more collectivistic than the samples included in the present study (Mak et al., 2014). Finally, the large Greek and Cypriot samples enable greater statistical power to generalize the findings to the wider adolescent population across the two countries and paves the way for future studies.

Despite the novelty of the study and the above-mentioned strengths, the present study is not without some limitations. Although studies have suggested that there are no statistically significant differences between traditional methods and Internet-based methods of data collection, differences in psychometric properties, such as factor loadings, have been found (Riva et al., 2003; Meade et al., 2007). In turn, this would influence MI testing. As a result, MI testing across all four countries was not applicable. Secondly, although variations of individualism and collectivism were postulated to affect the non-invariance finding in study one, these variables were not directly assessed or controlled. As a result, this invites further research.

### Future Direction

Prospective studies should aim to remedy these limitations. Future cross-cultural MI studies should include vertical and horizontal variations of individualism and collectivism to empirically determine whether these variables influence measurement equivalence testing. Exploring the psychometric equivalency of the OFQ in other collectivistic and individualistic cultures would not only provide more understanding of the applicability of the OFQ in these cultures, but also illustrate the extent at which cultural variations can impact the psychometric testing of universally applied constructs of cyber-psychology, applicable both in the field of Internet innovations and IGD.

## Conclusion

The psychological concept of Flow has grown exponentially since its inception in research. In recent times, with the global adoption of Internet use in everyday life, a new frontier has emerged for Flow research. This is evident in general Internet use research (e.g., Chen et al., 1999) and in more specific Internet contexts such as IGD (e.g., Stavropoulos et al., 2013). Clinicians also benefit from this expanding body of research in their practice. Namely, in the context of IGD, an understanding of the Flow mechanisms that may pull individuals into unhealthy behavioral patterns can play a vital role in therapy. While Flow research is fruitful, there is a lack of psychometrically sound instruments to measure Flow, especially, instruments that are reliable and valid in the presence of different cultural effects. This latter point is crucial because Internet use and Flow are considered to be universally experienced.

## Data Availability

The datasets generated for this study are available on request to the corresponding author.

## Ethics Statement

All procedures performed in this study involving human participants were in accordance with the ethical standards of the Research Ethics Committee from Federation University, Palo Alto University, University of Athens and the Cypriot Youth Organization. Informed consent was obtained from all participants and in the case of participants under the age of 18, written parental informed consent was obtained.

## Author Contributions

EH, VS, and AA contributed to the literature review, hypotheses formulation, data analyses, the structure and sequence of theoretical arguments, and manuscript revisions. MC and RG contributed to the structure and sequence of theoretical arguments and manuscript revisions. VS, CB, and SP contributed to the data collection.

## Conflict of Interest Statement

The authors declare that the research was conducted in the absence of any commercial or financial relationships that could be construed as a potential conflict of interest.

## Footnotes

1 The WLSMV indicator was used here because the potential answers of the OFQ were bivariate (dichotomous) and therefore polychronic matrices needed to be applied to calculate the χ^2^ value of the absolute fit of the model.

2 The recommended threshold for acceptable model fit for CFI is anything greater than 0.95 (Hu and Bentler, 1999).

3 For TLI, Hu and Bentler (1999) suggested anything over 0.95 to be acceptable fit.

4 For RMSEA, MacCallum et al. (1996) suggested 0.01 (excellent fit), 0.05 (good fit), and 0.08 (mediocre fit). However, other scholars suggested using 0.10 as the cut-off (Kenny, 2014).

5 Item 1 of the OFQ is as follows: “My mind isn’t wandering. I am not thinking of something else. I am totally involved in what I am doing. My body feels good. I don’t seem to hear anything. The world seems to be cut off from me. I am less aware of myself and my problems. My concentration is like breathing. I never think of it. I am really quite oblivious to my surroundings after I really get going. When I start, I really do shut out the whole world. Once I stop, I can’t let it back in again. I am so involved in what I am doing. I don’t see myself as separate from what I am doing. Have you ever encountered the situation indicated by any one of above paragraphs in your Web navigation and use?”).

## Appendix A: The Online Flow Questionnaire – English Version

                    Online Flow Questionnaire
Please read carefully the following paragraphs:
1. My mind isn’t wandering. I am not thinking of something else. I am totally involved in what I am doing. My body feels good. I don’t seem to hear anything. The world seems to be cut off from me. I am less aware of myself and my problems. My concentration is like breathing. I never think of it. I am really quite oblivious to my surroundings after I really get going. When I start, I really do shut out the whole world. Once I stop, I can let it back in again. I am so involved in what I am doing. I don’t see myself as separate from what I am doing. Have you ever encountered a situation indicated by any one of above paragraphs when navigating online?
2. When navigating online, have you ever experienced the feeling of ‘time going too fast’?
3. Have your ever experienced the feeling of enjoyment when navigating online?
4. Have you ever experienced the feeling of ‘positive challenge’ when navigating online?
5. Have you ever experienced the feeling of ‘being controlled by something’ when navigating online?

## Appendix B: The Online Flow Questionnaire – Greek Version

                    EΡΩΤΗΜΑΤΟΛΟΓΙΟ ΔΙΑΔΙΚΤΥΑΚΗΣ ΔΡΑΣΗΣ/ ΡΟΗΣ

Παρακαλώ διάβασε προσεκτικά τις ακόλουθες παραγράφους:
1. Το μυαλό μου δεν διασπάται. Δεν σκέφτομαι τίποτε άλλο. Είμαι ολοκληρωτικά απορροφημένος σε αυτό που κάνω. Αισθάνομαι καλά το σώμα μου. Δε μου φαίνεται να ακούω κάτι. Ο κόσμος μοιάζει αποκομμένος από μένα. Είμαι αποστασιοποιημένος από τον εαυτό και τα προβλήματά μου. Η συγκέντρωση είναι όπως η ανάσα μου. Δεν τη σκέφτομαι καθόλου. Είμαι αρκετά απαθής σε ότι πραγματικά με περιβάλλει, αφού ξεκινήσω. Όταν ξεκινάω, είναι σαν όλος ο κόσμος να κλείνει. Μόλις σταματήσω, επιστρέφω. Είμαι τόσο εμπλεγμένος σε αυτό που κάνω, που δεν μπορώ να με διαχωρίσω από αυτό. Έχεις ποτέ βρεθεί σε μία κατάσταση, από αυτές που περιγράφονται στις παραπάνω παραγράφους, κατά τη διάρκεια της περιήγησής σου στο διαδίκτυο.
3. Κατά την περιήγησή σου στον ιστό, αισθάνθηκες ποτέ το χρόνο να κυλάει πολύ γρήγορα;
5. Είχες ποτέ το αίσθημα της ικανοποίησης, κατά την περιπλάνησή σου στο διαδίκτυο;
7. Είχες ποτέ το συναίσθημα μίας θετικής πρόκλησης κατά την περιήγησή σου στον ιστό;
9. Ασθάνθηκες ποτέ ότι κάτι σε ελέγχει, κατά την περιήγησή σου στον ιστό;
